# Investigation of Molecular Details of Keap1-Nrf2 Inhibitors Using Molecular Dynamics and Umbrella Sampling Techniques

**DOI:** 10.3390/molecules24224085

**Published:** 2019-11-12

**Authors:** Ashwini Machhindra Londhe, Changdev Gorakshnath Gadhe, Sang Min Lim, Ae Nim Pae

**Affiliations:** 1 Convergence Research Center for Diagnosis, Treatment, and Care system of Dementia, Korea Institute of Science and Technology, Seoul 02792, Korea or 615502@kist.re.kr (A.M.L.); gadhe.changdev@gmail.com (C.G.G.); smlim28@kist.re.kr (S.M.L.); 2 Division of Bio-Medical Science & Technology, KIST School, Korea University of Science and Technology, Seoul 02792, Korea

**Keywords:** Keap1-NRF2 inhibitors, PPI inhibition, molecular modeling, MD simulations, US simulation, binding free energy

## Abstract

In this study, we investigate the atomistic details of Keap1-Nrf2 inhibitors by in-depth modeling techniques, including molecular dynamics (MD) simulations, and the path-based free energy method of umbrella sampling (US). The protein–protein interaction (PPI) of Keap1-Nrf2 is implicated in several neurodegenerative diseases like cancer, diabetes, and cardiomyopathy. A better understanding of the five sub-pocket binding sites for Nrf2 (ETGE and DLG motifs) inside the Kelch domain would expedite the inhibitor design process. We selected four protein–ligand complexes with distinct co-crystal ligands and binding occupancies inside the Nrf2 binding site. We performed 100 ns of MD simulation for each complex and analyzed the trajectories. From the results, it is evident that one ligand (1VV) has flipped inside the binding pocket, whereas the remaining three were stable. We found that Coulombic (Arg483, Arg415, Ser363, Ser508, and Ser602) and Lennard–Jones (Tyr525, Tyr334, and Tyr572) interactions played a significant role in complex stability. The obtained binding free energy values from US simulations were consistent with the potencies of simulated ligands. US simulation highlight the importance of basic and aromatic residues in the binding pocket. A detailed description of the dissociation process brings valuable insight into the interaction of the four selected protein–ligand complexes, which could help in the future to design more potent PPI inhibitors.

## 1. Introduction

In the last decade, several efforts have been made to find novel non-covalent inhibitors for the Keap1-Nrf2 pathway [1,2,3,4,5]. Nrf2 is a precursor for several defensive enzymes and antioxidants used against xenobiotics [6,7]. Accumulation of this species is a significant cause of neurodegenerative disease, cancer, diabetes, and others [8,9,10]. Keap1 is a sensor for reactive oxygen and reactive nitrogen species (ROS/RNS) [11]. Furthermore, it negatively regulates Nrf2 expression [12]. Inhibition of the protein–protein interaction (PPI) between these two proteins promotes the synthesis of antioxidant enzymes for cell protection [13]. The Kelch domain dimer binds with the DxETGE (Asp77-Glu82) and DLGex (Met17-Gln51) motifs of Nrf2 protein (Figure S1) [7,14]. The crystal structure of DxETGE and DLGex motif interactions with the Keap1-Kelch domain is available (Protein data bank (PDB) ID: 2FLU, 3WN7). Based on the Kelch-Nrf2 (DxETGE) motif interactions Jiang and co-workers divided the Kelch substrate-binding pocket into five sub-pockets, P1–P5 (Figure S1) [3]. Keap1-Nrf2 inhibitors, which bind at the five sub-pocket binding sites of the Kelch domain, can interfere with the binding of the ETGE and DLG motifs with Keap1. The journey of PPI inhibition initially started from the investigation of covalent inhibitors, which were having an off-target effect. Therefore, non-covalent inhibitors were designed by mimicking the DxETGE and DLGex peptide [15,16,17]. These lengthy peptides inhibitors then gradually take over by small non-covalent inhibitors [18,19]. Non-covalent inhibitors offer several advantages over covalent inhibitors in terms of improved selectivity and reduced toxicity. Various approaches, including high-throughput screening, fragment-based drug design, virtual screening, and lead optimization, have been used to develop novel non-covalent inhibitors [1,4,18,19,20]. Several X-ray crystal structures of small molecules complexed with the Keap1-Kelch domain are available in the Protein Data Bank (PDB).

The binding site consists of Arginine residues (Arg380, Arg415, and Arg483), and these electrostatic interactions give negative ionizable features in interaction-based pharmacophore design approach [21,22]. Virtual screening using such a pharmacophore may not give sufficient output. Several non-covalent inhibitors have acid-functional groups, which might affect the cell penetration. To overcome this problem, researchers are now focusing their studies on the design of non-acid group containing inhibitors [23,24].

Docking study is a commonly used technique for the prediction of protein–ligand interactions. In docking study, protein is rigid and ligand would be flexible to sample various binding confirmations. However, for prediction of accurate binding, receptor and ligand flexibility is essential. Therefore, to understand the dynamics behind keap1-Nrf2 inhibitors, we implemented advance molecular simulation techniques, such as molecular dynamics (MD) and umbrella sampling (US) techniques. A detailed analysis of the simulation trajectories could shed light on critical parameters necessary for strong and stable interactions.

**Figure 1:**
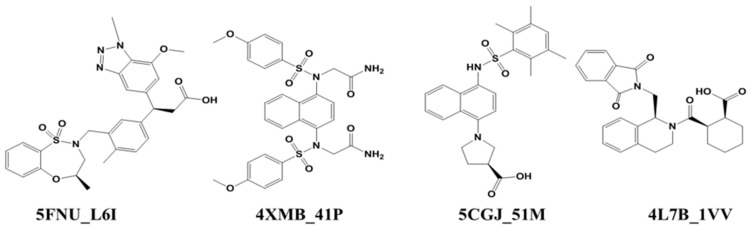
Co-crystal ligand structures of 5FNU, 4XMB, 5CGJ, and 4L7B.

MD simulation is effective and accessible techniques for understanding the macromolecular structure and functions [25]. Several protein targets and their structural rearrangements have been successfully studied using this tool [26,27,28]. We performed molecular dynamics (MD) simulations and calculated the binding free energy of four selected co-crystal ligands using the US technique. For this study, we selected four crystal structures from the PDB: 5FNU, 4XMB, 5CGJ, and 4L7B (Figure S2) [23,29,30,31]. The 2D structures of the corresponding co-crystal ligands 5FNU_L6I, 4XMB_41P, 5CGJ_51M, and 4L7B_1VV shown in Figure 1. The selected ligands have diversity in their scaffolds and structural composition, as well as binding pocket occupancy, and activity (Table 1, Figures S2–S3). Ligand L6I has the highest activity, with an IC_50_ value of 15 nM, followed by 41P with an IC_50_ of 61 nM. The two other co-crystal ligands, 51M and 1VV, have IC_50_ values of 0.14 µM, and 2.3 µM, respectively. Despite different resolutions, every protein–ligand complex has given a similar time and system for simulation. Therefore, both ligand–protein will get a sufficient time to adjust concerning each other and then achieve their energy minima [32].

As previously reported, the co-crystal pose is not the only pose that a molecule can adopt inside the binding pocket; instead, it is the conformation most suitable for the crystallization process [33,34]. This implies that binding is a dynamic process where the molecule can organize/fit in multiple confirmations inside the binding pocket [35]. Therefore, before the US study, MD simulations performed to understand the stability of the ligand conformations inside the Kelch domain. Overall, by combining MD and US simulation, we performed in total 0.8 μs of simulation. MD simulations analyzed by several techniques including root mean square deviation (RMSD), the number of hydrogen bonds (H-bond) analysis, principal component analysis (PCA), interaction energy calculation, and determination of the free energy landscapes, for detailed information of each protein–ligand complex.

Our research output sheds light on the several ligand–protein interaction parameters that could be considered in the development of novel non-covalent Keap1-Nrf2 inhibitors. Instead of randomly designing the molecules, researchers should first simulate and understand the movement of ligands. Then unstable part or substituents can modified by calculating the LJ-SR and Coul-SR energies with targeted residues. Interaction energy of each ligand with particular amino acids might play a critical role in ligand stability inside the binding pocket. Particularly, we have observed a crucial role for the Arg483 residue. Although all four selected ligands interacted with Arg483 during the 50 ns MD simulations, their individual Coulombic and Lennard–Jones interaction energy values differ, with the most favorable energetics being those for the most active 5FNU_L6I ligand. Repeated trials using simulation-based analysis approach can improve the success rate in the design of keap1-Nrf2 inhibitors.

## 2. Results

### 2.1. Ligand–Protein Interactions Analysis

#### 2.1.1. Interaction before MD Simulation

We first analyzed the protein–ligand complex interactions of the selected four ligands using Discovery Studio client 2018 [36]. Their initial interactions with the Kelch domain are described in detail in the Supplementary Information.

#### 2.1.2. Docking Study

The docking study was performed using Schrödinger software [Schrödinger LLC, NY]. We selected four crystal structures aligned over each other (Figure S4A). Significant differences were observed in Asn382, Arg380, Arg415, and Arg483 side-chain orientation. L6I, 41P, 51M, and 1VV ligands docked in its discrete protein structures. The docking score of the topmost binding pose and RMSD concerning the crystal structure’s ligand-binding orientation is shown in Figure S4B and Table S1.

### 2.2. MD Simulation Results

**Figure 2:**
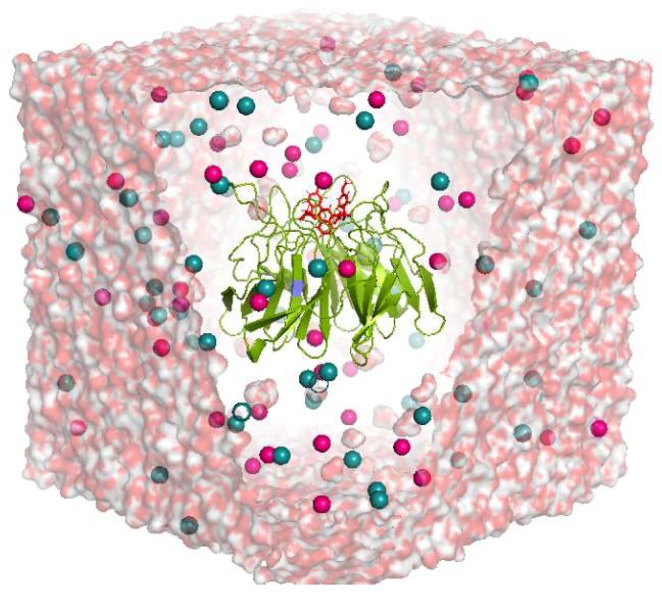
System prepared for molecular dynamics simulation. Protein–ligand complex kept at the center of the box. The 20 Å box is solvated and neutralized with sodium and chloride ions, shown as blue and pink spheres. Water shown in transparent surface representation. The protein is shown in green-colored cartoon representation and the ligand with red-colored stick format.

We selected four crystal structures to simulate using the Gromacs software version 5.0.6 [37]. Initially, we performed 50 ns simulation for each complex. After an analysis of 50 ns simulation results, we further extended the simulation for 100 ns. The simulation system was prepared using protein–ligand complex in a box of water along with sodium and chloride ions for neutralization of the system, as shown in Figure 2. Details of system preparation are included in the Material and Methods section. Stability of protein–ligand complexes during simulation period was analyzed by RMSD of the protein backbone and ligand structure. Further, MD simulation trajectories were used to investigate several criteria such as H-bonds analysis, residue interaction energy, principal component analysis (PCA), and free energy landscape analysis. Results obtained from each parameter are described here in detail.

#### 2.2.1. RMSD Calculation

**Figure 3:**
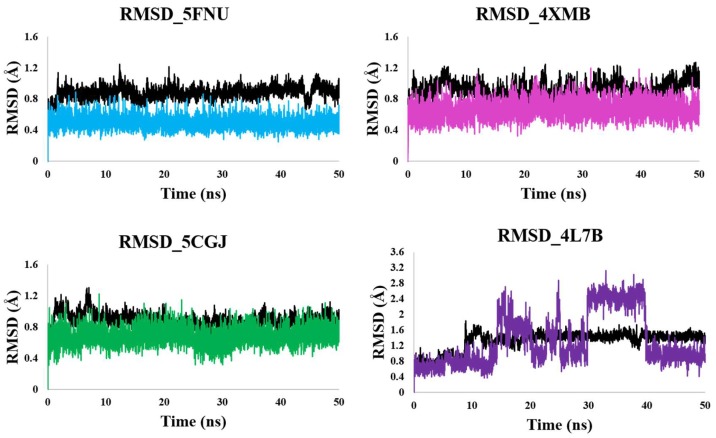
RMSD of ligand and protein backbone obtained from 50 MD simulation. RMSD of 5FNU_L6I, 4XMB_41P, 5CGJ_51M, and 4L7B_1VV shown. RMSD of protein backbone shown in black color. RMSD of L6I, 41P, 51M and 1VV ligands are shown in cyan, magenta, green, and purple respectively.

To evaluate the stability of each protein–ligand complex, the RMSD of the protein backbone and ligand were calculated during a 50 ns MD trajectory, as shown in Figure 3. The RMSDs showed that the 5FNU, 4XMB, and 5CGJ protein–ligand complexes were stable, but that of 4L7B was not. For the three stable complexes, the RMSD was less than 1 Å. This indicates that the initial ligand-backbone contacts remained intact during the simulation.

In case of 4L7B, the ligand showed multiple binding orientations. The ligand re-equilibrated three times during the 50 ns simulation (Figure 3). We also observed the flipping of the ligand orientation. Around 15 ns, the RMSD of the 1VV ligand increased by 2 Å, and the corresponding flipped orientation, as shown in Figure 5A in pink. Initially, the 1VV ligand occupied the P2, P3, and P5 pockets (Figure S2D). The ligand’s acid group formed a H-bond interaction with Arg415 (Figure S3). At 15 ns, the isoindole and cyclohexane groups flipped over each other (Figure 5A). The cyclohexane group shifted out of the P2 pocket, losing its acid-group-mediated interaction with Arg415 and remaining outside of the P5 pocket, without strong interactions. The isoindole group initially engaged in H-bonding and π–π-static contact with Ser602 and Tyr572 in the P5 pocket (Figure S2D). Flipping of this ring resulted in π-static interactions with Tyr334. The dihydro-isoquinoline group shifted from P3 to P5, making new contact with Phe577 (Figure 5A).

Subsequently, the RMSD rose by 2 Å at 30 to 40 ns. The 1VV ligand mainly interacted with aromatic residues like Tyr334, Phe577, and Tyr572 during this time (Figure 5B). The ligand oriented in such a way that the cyclohexane group remained on the upper side of the five sub-pockets of the Kelch domain binding site. Finally, the RMSD dropped to 1 Å ± 0.2 Å and oscillated during the 40–50 ns simulation period (Figure 3). The ligands held inside the pocket by two tyrosine residues, Tyr572 and Tyr334, forming H-bond and π–π stacking interactions (Figure 5C).

**Figure 4:**
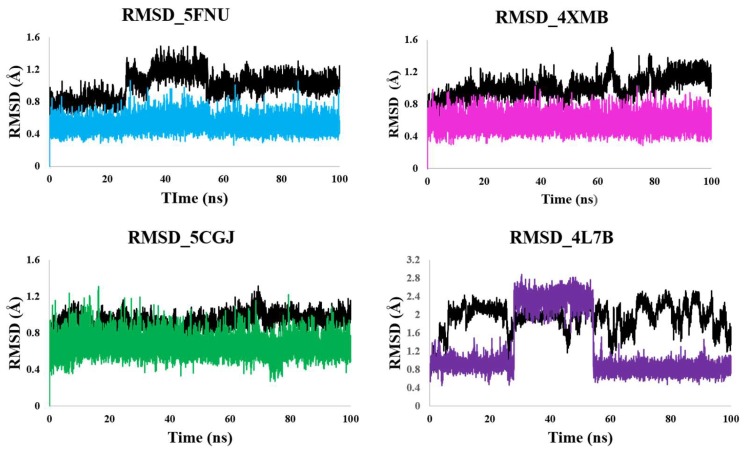
RMSD of ligand and protein backbone obtained from 100 ns MD simulation. RMSD of 5FNU_L6I, 4XMB_41P, 5CGJ_51M, and 4L7B_1VV shown. RMSD of protein backbone shown in black. RMSD of L6I, 41P, 51M and 1VV ligands shown in cyan, magenta, green, and purple respectively.

**Figure 5:**
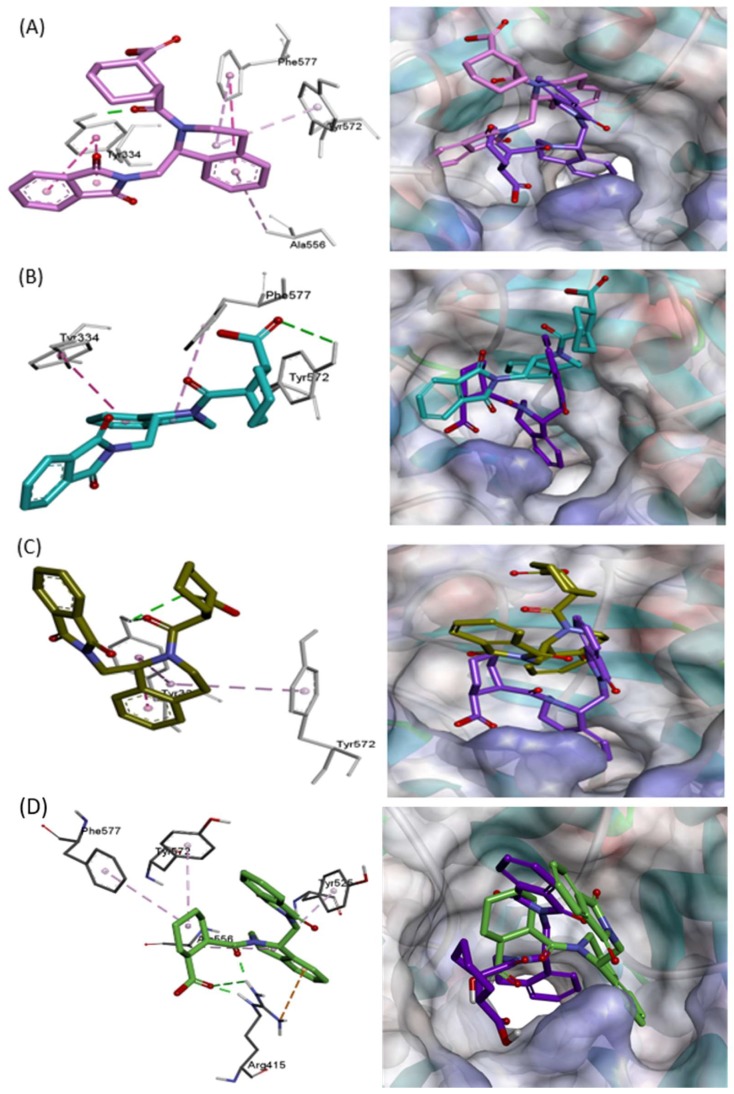
1VV ligand flipped and change orientation during MD simulation. Ligand–protein interactions at 15 ns (**A**), 35 ns (**B**), 45 ns (**C**), and 70 ns (**D**) shown by pink, cyan, deep olive, and green respectively. Overlapped co-crystal ligand orientation shown in purple.

We extended the simulation for 100 ns and obtained RMSD graphs shown in Figure 4. 5FNU, 4XMB, and 5CGJ complexes has shown stable RMSD. In case of 4L7B complex, backbone RMSD reaches to ~2.4 Å. 1VV ligands RMSD increase and reach to ~2.6 Å. After 60 ns ligand RMSD drop down and achieved stable RMSD at ~0.8 Å. Around 70 ns ligand orientation shown in Figure 5D. At this point, Arg415 holds the ligand by three H-bond interactions. Tyr525, Ala556, Tyr572, and Phe577 residues has only week hydrophobic contacts with ligand. When we analyzed the trajectories using VMD software, we observed that after 70 ns 1VV ligand frequently jumped out from the Kelch domain, which means it shows interactions with only Arg415. Therefore, from RMSD analysis it is clear that 1VV ligand-binding orientation changed over the simulation time.

#### 2.2.2. Hydrogen Bond Analysis

**Figure 6:**
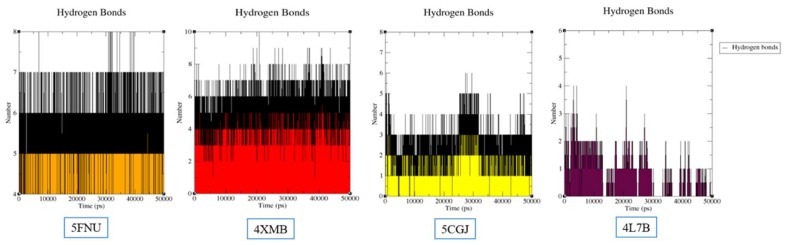
Assessment of number of H-bonds during 50 ns MD simulations.

The number of H-bonds was investigated using Gromacs g_hbond utility. The numbers of H-bonds between each ligand and protein of four selected crystal structures were analyzed using 50 ns simulation trajectories showed in Figure 6. This analysis shows that ligand-binding affinity will change as the number of H-bonds change. Four complex’s 5FNU_L6I (IC_50_ = 15 nM), 4XMB_41P (IC_50_ = 61 nM), 5CGJ_51M (IC_50_ = 0.14 µM), 4L7B_1VV (IC_50_ = 2.3 µM) has average number of 7–8, 8–10, 5–6 and 3–4 H-bonds, respectively. Nanomolar compounds showed a higher number of H-bonds than micromolar active compounds. Surprisingly, among L6I and 41P ligands, highest H-bonds were achieved by 41P. These findings also support our further binding free energy calculation results where 41P has shown the highest binding free energy.

L6I ligand showed an average of seven H-bonds continuously. This output will assist the stability of ligand with no changes in RMSD in the simulation period. 51M ligand showed a maximum of 5–6 H-bonds. At the end of the simulation, this value decreases to four, which is lower than L6I and 41P. This result correlates with 51M ligands lower activity as compared to L6I and 41P. In the 4L7B crystal structure, number of H-bonds are 3–4, and as the simulation progresses, this number further decreases and reaches a maximum of one H-bond. At 45 ns simulation, we have shown in Figure 5C, that, 1VV has only one H-bond interaction with Tyr334.

Similar trend of H-bond analysis was observed at the end of 100 ns simulation (Figure S5). 4XMB and 5FNU has shown highest H-bond interactions. At the beginning of 5CGJ simulation 5–6 H-bonds were observed which dropped down after 20 ns. At the end of simulation 5CGJ complex showed 3–4 H-bonds. In case of 4L7B complex, during the first half of the simulation it showed 3–4 H-bonds and this number further decreased in the second half of the simulation to 2–3 H-bonds.

The overall H-bond analysis clearly showed that the number of H- bond interactions contributed to the constant RMSD of 5FNU, 4XMB, and 5CGJ crystal structure during the simulation period. Whereas in 4L7B, a decrease in the initial number of H-bonds reflects lower stability inside the binding pocket.

#### 2.2.3. Residue Interaction Energy

Throughout the MD trajectory, the interaction energy of each ligand with surrounding protein residues within 4 Å was calculated. Residue interaction energy for 50 ns and 100 ns are shown in Table 2 and Table S2, respectively. The interaction energies are given in kJ/mol. Two types of short-range potential calculated: Lennard–Jones short-range (LJ-SR) and Coulombic short-range (Coul-SR) potential. We calculate the total sum of LJ-SR and Coul-SR potential of 4 Å residues of ligands. For 100 ns simulation, residue interaction energy results are shown in Table S2. The obtained sum of LJ-SR and Coul-SR potential are in line with the experimental activity of each ligand.

##### 5FNU

The L6I ligand (IC_50_ = 15 nM) made firm contacts with the Arg483 (H-bond and electrostatic interactions, Figure S3) and showed a higher Coul-SR interaction energy (−173.22 kJ/mol) than the other three ligands. L6I was fixed in the P3 pocket (Arg415) (Figure S2A) by hydrophobic interactions of the methyl phenyl ring, and therefore showed high LJ-SR interaction energy of −25.36 kJ/mol. Simultaneously, Arg415 shows electrostatic interactions with the carbonyl group of ligand but interactions seem to be negligible (4.019 kJ/mol). Gly530 in the P4 pocket formed an H-bond with the nitrogen of the triazole ring and showed strong Coul-SR interaction energy of −29.59 kJ/mol. By visualization in Discovery Studio, we have not observed H-bonds mediated by the serine residues Ser508, Ser602, and Ser555, although Ser363 showed a weak carbon-hydrogen bond. During the MD simulation, the serine residues changed orientation in order to establish strong H-bonds with the L6I ligand. Ser508, Ser602, Ser555, and Ser363 showed strong Coul-SR interaction energies of −59.40 kJ/mol, −29.12 kJ/mol, −13.48 kJ/mol, and −10.45 kJ/mol, respectively. Furthermore, the aromatic residues involved in π–π stacking interaction showed strong LJ-SR interaction energies of −22.08 kJ/mol for Tyr334 and −34.91 kJ/mol for Tyr525, followed by Tyr572 with a π-alkyl contact having an energy of −14.14 kJ/mol. The substituent inside the P3 pocket also made hydrophobic contact with Ala556 with the −11.81 kJ/mol LJ-SR interaction energy.

After 100 ns simulation analysis, similar results observed (Table S2). Total LJ-SR and Coul-SR energies for 50 ns and 100 ns is −506.67 kJ/mol and −498.34 kJ/mol, respectively. Considering the result of 50 ns (LJ-SR: −171.79, Coul-SR: −334.88) and 100 ns (LJ-SR: −152.89, Coul-SR: −345.45) simulation, it is clear that contribution of Coul-SR potential in 5FNU complex was greater than LJ-SR potential.

##### 4XMB

The 4XMB co-crystal ligand, 41P (IC_50_ = 61 nM), occupied the five sub-pockets of the Kelch domain, similarly to the L6I ligand. Inside the P2 and P3 pockets Arg415 was involved in H-bond, electrostatic, and hydrophobic contacts (Figures S2B and S3), resulting in a high value for both the LJ-SR (−24.87 kJ/mol) and Coul-SR (−43.66 kJ/mol). As the 41P ligand did not have an electrostatic interaction with Arg483, compared with the L6I ligand, its Coul-SR interaction energy was negligible. Ser363, Ser508, Ser602, and Asn414 were involved in H-bond interaction and showed high values for Coul-SR interaction energy of −22.68 kJ/mol, −18.12 kJ/mol, −17.11 kJ/mol, and −22.40 kJ/mol respectively. Ala556 held the naphthalene ring in the P3 pocket by π-alkyl and π-σ interactions, giving LJ-SR interaction energy of −15.60 kJ/mol. The Discovery Studio interaction viewer detected no interaction with Tyr334, Tyr525, and Tyr572 (Figure S2B), but during certain time intervals of the MD simulation, such interactions were visible. Tyr334, Tyr525, and Tyr572 showed LJ-SR interaction energies of −15.32 kJ/mol, −19.3 kJ/mol, and −16.75 kJ/mol, respectively. A Phe577 mediated π–π stacking interaction was observed initially, but its interaction energy (8.85 kJ/mol) was unfavorable than other tyrosine residues.

Results obtained after 100 ns analysis were similar to 50 ns simulation data (Table S2). Total LJ-SR and Coul-SR energies for 50 ns and 100 ns is −343.56 kJ/mol and −344.82kJ/mol, respectively. By analyzing the result of 50 ns (LJ-SR: −203.58 kJ/mol, Coul-SR: −139.98 kJ/mol) and 100 ns (LJ-SR: −201.51 kJ/mol, Coul-SR: −143.31 kJ/mol) simulation it is clear that contribution of LJ-SR potential in 4XMB complex is greater than Coul-SR potential.

##### 5CGJ

The 51M (IC_50_ = 0.14 µM) ligand occupied mainly the P1, P3, and P5 pockets (Figure S2C). A H-bond with the crucial Arg483 (Figure S2C) resulted in a −39.09 kJ/mol Coul-SR potential energy. In the P3 pocket, Arg415 and Ala556 firmly bound the naphthalene group by LJ-SR potential energies of −29.07 kJ/mol and −14.91 kJ/mol respectively. Ser602 and Ser508 had intense H-bonds resulting in strong Coul-SR potential energies of −21.23 kJ/mol and −11.05 kJ/mol, respectively. Another serine, Ser363, was involved in H-bond interaction but showed lower values for both LJ-SR and Coul-SR potential energy (−5.54 kJ/mol and −6.43 kJ/mol). This result indicates that the Ser363 interactions were not stable during the simulation period. Tyr334 showed strong π–π stacking, π–σ, and π–alkyl contacts with a −21.41 kJ/mol LJ-SR potential energy. Discovery Studio also showed π-alkyl (hydrophobic) interactions of Phe577 and Tyr572 resulted in −7.69 kJ/mol and −10.79 kJ/mol LJ-SR potential energies.

After analysis of 50 ns simulation data, we extended the simulation to 100 ns. Results obtained after 100 ns simulation analysis (Table S2) are very similar to 50 ns data. Total LJ-SR and Coul-SR energies for 50 ns and 100 ns is −250.34 kJ/mol and −230.06 kJ/mol, respectively. By analyzing the result of 50 ns (LJ-SR: −175.77kJ/mol, Coul-SR: −74.57 kJ/mol) and 100 ns (LJ-SR: −166.04 kJ/mol, Coul-SR: −64.02 kJ/mol) simulation, it is clear that contribution of LJ-SR potential in 5CGJ complex is greater than Coul-SR potential.

##### 4L7B

1VV (IC_50_ = 0.75 µM) did not occupy the binding pockets entirely. In the initial pose of 1VV, we observed a H-bond interaction of the ligand’s acid group with Arg415 and Asn414 inside the P2 pocket (Figure S2D, Figure S3), which is not displayed in the PDB2D diagram. 1VV ligand showed fluctuations in RMSD until 50 ns. We will therefore first discuss 50 ns residue interaction energy results. The Coul-SR potential energy for Arg415 and Asn414 was −10.12 kJ/mol and −4.11 kJ/mol, respectively. Arg415 showed hydrophobic contacts with the isoquinoline ring gave rise to LJ-SR energy of −7.44 kJ/mol. Compared with other ligands, 1VV showed lower interaction energy with the crucial Arg415 residue, which might be the reason for its lower activity. Ala556 lost its hydrophobic interaction after 20 ns and showed a −3.28 kJ/mol LJ-SR potential energy. Tyr334 in the co-crystal pose showed only π-alkyl contact with the cyclohexane ring, but during the simulation time, the ligand flipped and changed its conformation, so Tyr334 showed several π–π stacking and H-bond interactions (Figure 4). Tyr334 in the interaction analysis showed moderate LJ-SR and Coul-SR potential energies (−17.44 kJ/mol and −18.71 kJ/mol). In the co-crystal pose, Ser602 shown H-bond with the oxygen of the isoquinoline ring, but this residue had a lower value for the Coul-SR (−3.46 kJ/mol) potential energy, because this interaction was disrupted after 15 ns when the ligand flipped (Figure S4A,B). Tyr572 stayed near the ligand throughout the simulation and showed several π–alkyl and π–π stacking interactions with a moderate (−13.48 kJ/mol) LJ-SR potential energy. Up to 50 ns simulation analysis contribution of LJ-SR potential was more than Coul-SR potential.

As we extended the simulation to 100 ns, RMSD of 1VV ligand reduced after 50 ns and remained constant until the end of the simulation. However, ligand interactions before and after 50ns simulation were not similar. Therefore, the differences in total LJ-SR and Coul-SR energies for 50 ns (−125.24 kJ/mol) and 100 ns (−227.2 kJ/mol) were observed (Table S2). In the first half of the simulation, ligands interactions with Arg415 diminished; however, after 50 ns ligand again showed strong polar contacts with Arg415. Therefore, at the end for 100 ns simulation LJ-SR and Coul-SR potential of Arg415 increased to −22.24 kJ/mol and −81.kJ/mol, respectively. Simultaneously contribution of Coul-SR potential of Asn414 (−20.48 kJ/mol) and LJ-SR of Tyr525 (−9.53 kJ/mol) also increased. At the end of the 100 ns simulation, Coul-SR (−113.72 kJ/mol) and LJ-SR (−113.48 kJ/mol) potentials showed equal contribution for ligand–protein binding.

The overall interaction energy of the 1VV ligand with surrounding residues was modest. 1VV lacked interactions with several crucial residues, such as Arg483, Tyr525, and Ser555. 1VV was not stable inside the binding pocket during the MD simulation, which is the probable reason for its lower activity.

Apart from serine, arginine, tyrosine, and phenylalanine; several glycine residues (Gly364, Gly462, Gly509, Gly530, and Gly603) were involved in H-bond, hydrophobic, and van der Waals interactions.

#### 2.2.4. Principal Component Analysis

**Figure 7:**
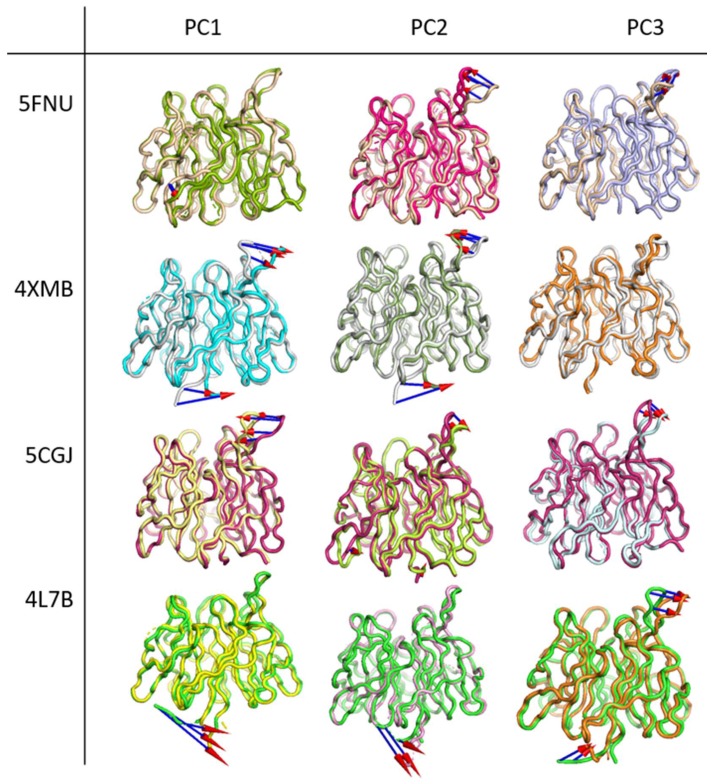
Principal component analysis. Eigenvectors from 50 ns MD trajectories depicting the movement of Cα atoms in four crystal structures. The initial position of the protein backbone shown in wheat, gray-white, pink, and green for 5FNU, 4XMB, 5CGJ, and 4L7B, respectively. The movements of flexible parts of the protein shown with arrows with red heads and blue tails.

The conformation changes in the protein backbone were ascertained by principle component analysis (PCA). The first three PCs predicted the majority of the motion of the protein backbone from the MD trajectories. PCA analysis shows that the first three eigenvectors account for 19.5%, 36.88%, 20.36%, and 48.19% of motion in 5FNU, 4XMB, 5CGJ, and 4L7B complexes, respectively. To visualize the movement of the backbone, the ‘Modevectors’ plugin in the PyMol software is used. A porcupine plot was generated by aligning the trajectories over the original protein structure. The movements of flexible parts of the protein shown with arrows with red heads and blue tails in Figure 7.

The arrow length shows the magnitude of the movement, and the cone shows the direction. This eigenvector shows that the main movements are those of the second blade and N-terminal part of the protein. The Arg380 and Asn382 residues on the second blade are involved in H-bond interactions with the ETGE motif (Glu82, Phe83) and DFGex motif (Leu23, Gln26, Asp27) of the Nrf2 peptide. These two amino acids fully occupy the P2 pocket of the Keap1-binding site. It is apparent from the PCA that the protein structure is very stable throughout the MD simulation and minimal movement occurs only at the second blade and C-terminal. Similar results obtained from 100 ns simulation. PCA analysis of 100 ns simulation shown in Figure S6. The 100 ns PCA analysis shows that the first three eigenvectors account for 22.85%, 29.90%, 11.35%, and 30.50% of motion in 5FNU, 4XMB, 5CGJ, and 4L7B complexes, respectively.

#### 2.2.5. Free Energy Landscape

**Figure 8:**
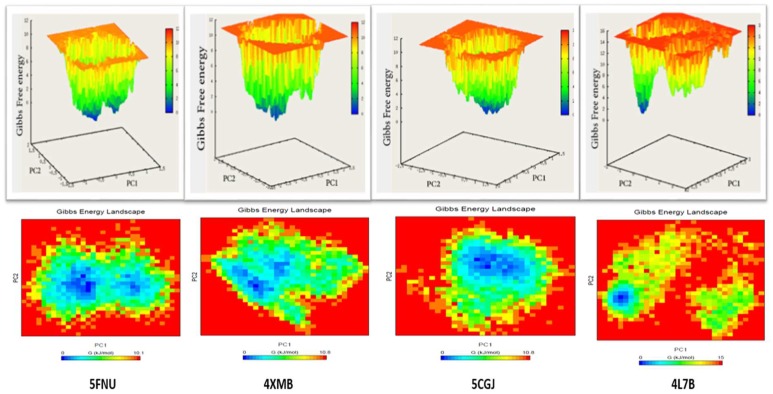
Free energy landscapes of four crystal structures during 50 ns MD simulation. 2D and 3D graphs projected on the first two principal components (PC1 + PC2). Blue spots indicate the energy minima.

In order to determine the low-energy basins (minima) explored during the simulation, we performed a free energy landscape (FEL) analysis. Both 2D and 3D graphs of the FEL were plotted using PC1 and PC2. Results of the free energy landscape for 50 ns and 100 ns are shown in Figure 8 and Figure S7, respectively. Each protein–ligand complex has a different pattern for the FEL. Dark blue spots indicate the energy minima and energetically favored protein conformations, and yellow spots reflect the unfavorable conformations. The shallow and narrow energy basin observed during the simulation revealed the low stability of the protein–ligand complex. In the case of 5FNU, two distinct deep and broad valleys were observed, while in 4XMB, two connected energy minima were seen. 5CGJ showed a cluster consisting of two energy basins close to each other. In these three cases (5FNU, 4XMB, 5CGJ), the initial Gibbs free energies were between 10.1 kJ/mol to 10.8 kJ/mol, but in 4L7B, the initial Gibbs free energy was higher (15 kJ/mol) than the other three complexes.

FEL obtained after 100 ns of simulation shown in Figure S7 As the 5FNU, 4XMB, and 5CGJ complexes has shown stable RMSD so their energy minimas are broader and clear. In the case of 4L7B, complex ligand and backbone both showed more RMSD deviations so single compact energy minima were not observed.

### 2.3. Umbrella Sampling (US) Results

**Figure 9:**
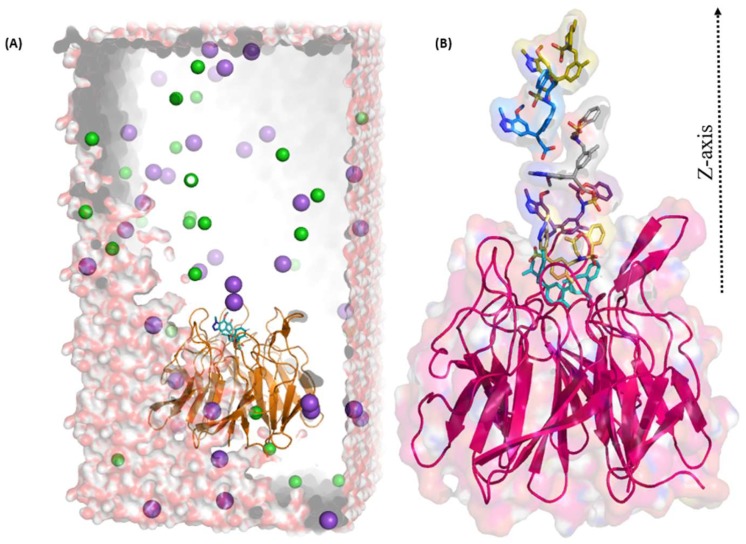
Umbrella sampling system and snapshots of the center-of-mass-pulling. (**A**) US system constructed using a box with a *z*-axis 12 nm in length. To pull the ligand in the *z*-axis direction, the *z*-axis was elongated. The protein is shown in yellow and the ligand in cyan. Sodium and chloride ions are shown as purple and green spheres respectively. The solvent is shown in surface representation. (**B**) Snapshots of the center-of-mass-pulling simulation. Protein is shown in magenta cartoon representation and ligands in stick format.

To calculate the binding free energy of the four MD simulated ligands, we used the US technique. The average structure from the last 10 ns of each simulation was selected for further US study. Systems were prepared using Gromacs software using similar parameters like MD except for the box size (Figure 9A). In order to pull the ligand out from the binding pocket, the box was elongated along the *z*-axis, as shown in Figure 9B. System preparation, ligand pulling, and US simulation steps are described in the Material and Method section. Each co-crystallized ligand was pulled along the *z*-axis with a constant force of 1000 kJ/(mol/nm) over 300 ps at the rate of 0.01 nm/ps. This pulling simulation generated 300 configurations. We used 401 configurations in total for the four crystal structures and each was simulated for 1 ns. Therefore, in total, 401 ns were simulated, in addition to the MD simulations that were performed to reach the equilibration stage of the potential of mean force (PMF) graphs.

The potential of mean force (PMF) was extracted from the US simulations. Weighted histogram analysis method (WHAM) was used to extract the PMF curves from the simulation trajectories. Several energy minima were obtained in the PMF graphs, each of which reflects a minimum energy conformation obtained after breaking some specific interactions between protein and ligand molecules. The binding free energy (ΔG) was calculated by taking the difference between the highest and lowest value of the PMF graphs (Table 3). The PMF graph has energy in kcal/mol on the *y*-axis, and the distance pulled in nm on the *x*-axis. The distance from the ligand was calculated from the center of mass of the protein. Our obtained binding free energies differed consistently between the molecules with micromolar and those with nanomolar activity. Most negative binding free energy value denotes a higher binding affinity of the ligand. The results obtained from the binding free energy calculations are discussed individually for each protein–ligand complex.

5FNU

**Figure 10:**
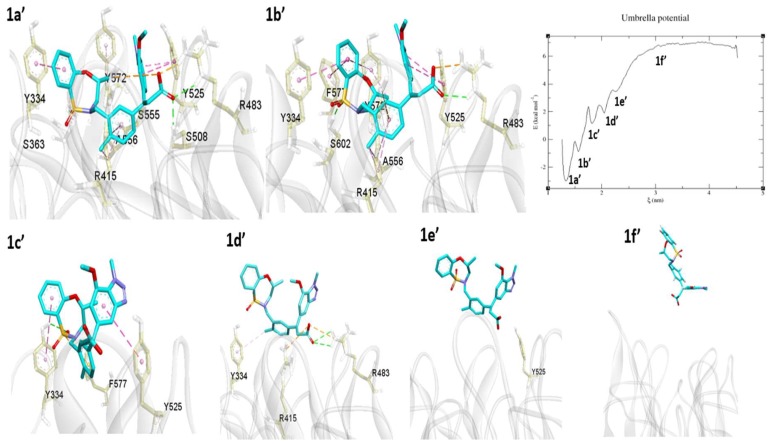
Unbinding pathway for L6I (5FNU) ligand. PMF graph obtained from US simulation of L6I shown in the right side. 1a’, 1b’, 1c’, 1d’ and 1e’ are the energy minima’s and 1f’ is the equilibrium stage obtained during the US. L6I ligand shown in cyan-color stick format. The protein is shown as solid ribbon and binding site residues in yellow color stick format. Interactions such as H-bonds, electrostatic, π–π stacking, π–σ, π–sulfur, and π–alkyl/alkyl are shown in green, orange, dark pink, purple, yellow, and light pink, respectively.

L6I co-crystallized ligand (IC_50_ = 15 nM) occupied the binding site with several interactions. From the interaction energy calculation, it is clear that the ligand had strong interactions with surrounding residues, especially Tyr334, Arg415, Arg483, Ser508, Tyr525, Gly530, Ser555, Ala556, Tyr572, and Ser602. The ligand and protein showed stable RMSD during the MD simulation, so the final snapshots were selected for US simulation. During the dissociation process, the L6I ligand showed five energy minima (Figure 10 1a’−1e’). 1a’ is the first minimum in the unbinding process. At this snapshot, interactions are almost the same as compared to crystal structure except for attractive electrostatic interactions of the acid group with Arg415.

1b’ is the second energy minimum, obtained after breaking the H-bonds between the ligand and Ser508, Ser555. Simultaneously, phenyl ring established π–π stacking interaction with Phe577 and Tyr572. In the next energy minimum, observed at the 1c’ position, several interactions, including those with Arg415, Arg483, Ala556, Tyr572, and Ser602, disappeared. Only three residues, Phe577, Tyr334, and Tyr525, still held the molecule. Further pulling reaches to the 1d’ energy minimum, where bonds with Tyr525 Phe577, were disrupted. During this process, the molecule started to come out from the binding cavity. At this point, the acid part of the ligand has H-bonds and attractive electrostatic interactions with Arg415 and Arg483. In addition, Tyr334 has weak π–alkyl contact with the methyl group. As the pulling simulation progressed, the ligand moved to the 1e’ energy minimum, by which time the molecule had lost most of the strong interactions and only Tyr525 remained in its vicinity, showing a van der Waals interaction. The molecule again pulled continuously, and PMF graph attained equilibration at around ~3 nm distance. At this position, the molecule was entirely unbound and became solvent-exposed. During this pulling process, the L6I ligand showed a −9.80 kcal/mol binding free energy.

4XMB

In the case of 4XMB ligand–protein complex, 41P ligand pulled 4.282 nm from the binding pocket. This unbinding process requires −13.48 kcal/mol energy to dissociate the ligand. Among the four tested compounds, 41P (IC_50_ = 61 nM) showed more negative binding free energy. As the 41P, ligand has amide, hydroxyl, and aromatic ring sub-structures. During the pulling simulation, it interacted with several amino acids, leading to an extended stay inside the binding pocket. In addition, in the H-bond analysis 41P ligand has shown highest 8–10 average H-bond interactions (Figure 6). These results are consistent and supportive of its highest binding free energy requirement.

**Figure 11:**
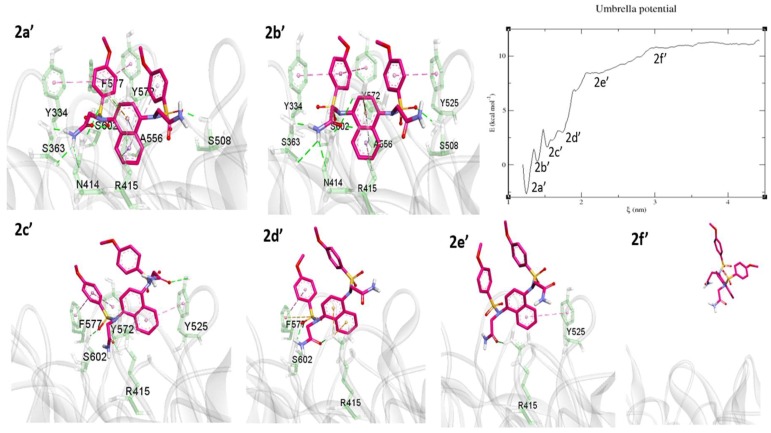
Unbinding pathway for 41P (4XMB) ligand. PMF graph obtained from US simulation of 41P shown in the right side. 2a’, 2b’, 2c’, 2d’ and 2e’ are the energy minima’s and 2f’ is the equilibrium stage obtained during the US. 41P ligand shown in magenta stick format. Protein shown as transparent ribbon and binding site residues in green stick format. Interactions such as H-bonds, electrostatic, π–π stacking, π–σ, π–sulfur, and π–alkyl/alkyl are shown in green, orange, dark pink, purple, yellow and light pink, respectively.

During the dissociation process, we have observed five energy minima (Figure 11 2a’–2e’). 2a’ is the first energy minimum in the binding process obtained after breaking the contacts with Ile461, Gly462, and Tyr525. The second energy minimum 2b’, was where the π-stacking interaction with Phe577 was broken. During the pulling process, 41P ligand breaks and make several bonds with surrounding residues. Therefore, overall more energy is needed to break these bonds, for example, breaking and remaking the interactions with Tyr525 at 2a’−2e’ position. At the 2c’ position H-bonds with Ser363, Asn414, and Ser508, and Ala556 interactions diminished. At the 2d’ position only Phe577, Ser602, and Arg415 hold the molecule in the pocket. Further pulling brought the ligand fully out from the binding pocket. The next energy minimum was observed at 2e’, where only two residues, Arg415 (with amide oxygen) and Tyr525 (with naphthalene ring), still hold the molecule through an H-bond and π–π stacking interaction, respectively. These two residues played a crucial role in holding the 41P ligand inside the pocket. These observations were consistent with our interaction energy calculation in the MD analysis, where Arg415 (LJ-SR = −43.66 kJ/mol, Coul-SR = −24.87 kJ/mol) and Tyr525 (LJ-SR = −19.39 kJ/mol) showed highest interaction energies. Around ~3 nm onwards (2f’), the PMF graph becomes equilibrated, and the ligand loses all interactions, including van der Waals interactions, and becomes completely solvent-exposed.

5CGJ

**Figure 12:**
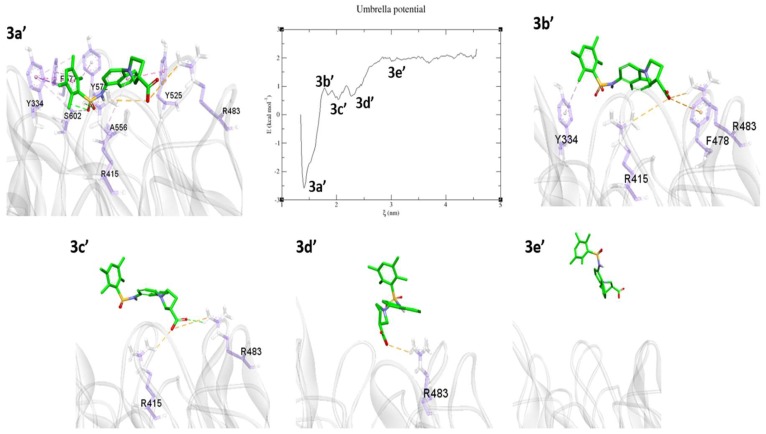
Unbinding pathway for 51M (5CGJ) ligand. PMF graph obtained from US simulation of 51M shown at the center. 3a’, 3b’, 3c’, and 3d’ are the energy minima’s and 3f’ is the equilibrium stage obtained during the US. 51M ligand shown in green stick format. The protein shown as transparent ribbon and binding site residues in purple stick format. Interactions such as H-bonds, electrostatic, π–π stacking, π–σ, π–sulfur, and π–alkyl/alkyl are shown in green, orange, dark pink, purple, yellow, and light pink, respectively.

The 5CGJ_51M complex (IC_50_ = 0.14 µM) showed a stable RMSD during the MD simulation. Hence, for further US study, we selected the average structure from last 10 ns of the MD trajectory. During the pulling simulation, at 3a’ minimum the 51M ligand has similar interactions as in the co-crystal structure except for an H-bond with Ser508 (Figure 12 3a’). To achieve the next energy minima 3b’, the PMF curve steeply rose to 1 kcal/mol. At 3b’, the interaction with several amino acids, including Ala556, Ser602, Tyr525, Tyr572, and Phe577, was diminished and weak π–alkyl interaction with Tyr334 observed via an alkyl group (Figure 12, 3b’). Arg415, Arg483, and Phe478 bound the acid moiety by electrostatic interactions. At the 3c’ minima achieved by breaking the contacts with Tyr334 and Phe478. The only acid part has H-bond and electrostatic interactions with Arg415, Arg483. Last minima observed at 3d’, tetra-methyl phenyl group pulled up and molecule ready to free from binding pocket showing only salt bridge contact with Arg483. From the 3d’ position, the PMF graph started to increase and equilibrated around ~2 kcal/mol. At the 3e’ position, the 51M ligand was completely solvent-exposed, with no interactions with protein residues.

4L7B

**Figure 13:**
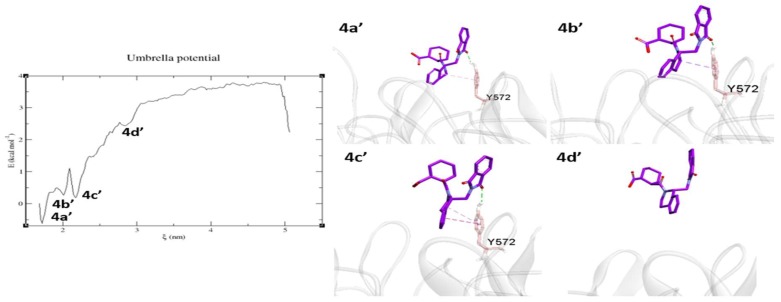
Unbinding pathway for 1VV (4L7B) ligand. PMF graph obtained from US simulation of 1VV shown in the left side. 4a’, 4b’, and 4c’ are the energy minima’s and 4d’ is the equilibrium stage obtained during the US. 1VV ligand shown in purple stick format. The protein shown as transparent ribbon and binding site residues in red stick format. Interactions such as H-bonds, electrostatic, π–π stacking, π–σ, π–sulfur, and π–alkyl/alkyl are shown in green, orange, dark pink, purple, yellow, and light pink, respectively.

The 4L7B co-crystal ligand 1VV (IC_50_ = 2.3 µM) before the MD simulation occupied the P2, P3, and P5 pockets of the Kelch domain. The interaction energy with the surrounding ligands was not strong enough to keep the molecule stable inside the binding pocket. As a result, the molecule showed a high RMSD of ~0.2 nm. Figure 13 shows the dissociation pattern for the 1VV ligand. 4a’ is the first energy minimum, at this position, the ligand made H-bonds and π–alkyl contacts with Tyr572 along with some van der Waals interactions with Phe577 and Tyr33 (not shown in the figure). Tyr572 seems to be crucial, as it is the only residue consistently interacted with the ligand at the 4b’ and 4c’ positions through H-bonds, π–σ, and π-stacking interactions. At 4d’, Tyr572 interaction was broken. At this point, the molecule was completely solvent-exposed. The Tyr572 residue showed a strong (−13.48 kJ/mol) LJ-SR potential in the MD simulation. From the US and MD study, it is clear that the aromatic residue Tyr572 contributed to ligand–protein interaction. The 1VV ligand showed a −4.35 kcal/mol binding free energy. This value is the least negative among the four tested molecules, so it signifies the weak ligand affinity towards Kelch domain.

## 3. Discussion

In this study, we aimed to calculate the binding free energy of four ligands of Keap1-Nrf2 non-covalent inhibitors, crystallized within the Keap1-Kelch domain. The objective was to analyses various parameters responsible for the stability of protein–ligand complexes and identify the crucial residues in the Keap1-Nrf2 binding pocket, which could be further exploited to design and develop potent PPI inhibitors. To execute our idea, we first performed an MD simulation study and then binding free energy of the four simulated co-crystallized ligands.

Currently, techniques such as MD simulation, metadynamics, and US are widely used to address many biological phenomena at the atomistic level. As we have directly taken crystals structures for MD simulation study, therefore 50 ns and then 100 ns simulation performed to equilibrate the system. In our study, we investigated in-depth and used several analysis techniques to understand the fundamental behind this ligand–protein interactions mechanism. From the simulation results, it is clear that all the ligand showed RMSD of less than 1.25 Å, except 4L7B. In case of 4L7B_1VV complex, the small size of ligand, partial binding pocket occupancy, and lower interaction energy with binding site residues led to wandering and shifting between various positions in the binding pocket; hence showed the RMSD fluctuations. Similar multiple binding modes for keap1-nrf2 inhibitors were reported by Satoh et al. using MD simulation and X-ray crystallography (3VNG, 3VNH) [38]. In their study, bound ligand tends to dissociate after 20 ns simulations. Stability and fluctuation of these ligands during MD simulation were supported by several H-bonds estimations. The lower number of H-bonds reflected the lower stability of ligand-binding conformations and vice versa.

From the MD simulations, we decided to determine the individual residue contributions to the interaction energy. Short-range Lennard–Jones (LJ-SR) and Coulombic (Coul-SR) potential energies were calculated (Table 2, Table S2). The sum of this LJ-SR and Coul-SR potential of surrounding 4 Å residues for the individual ligand is in line with the activity. After 100 ns, 5FNU (15 nM) showed highest value of −498.34 kJ/mol followed by 4XMB (61nM) with −344.82 kJ/mol potential. 5CGJ (0.14 µM) and 4L7B (0.75 µM) showed −230.06 kJ/mol and −227.2 kJ/mol, respectively. Arg483 shown highest Coul-SR potential with L6I than the remaining three inhibitors, reflecting its utmost importance for the strong electrostatic interaction, which may be responsible for L6I highest potency. In the case of 41P ligand, Arg415 made an immense contribution towards the LJ-SR and Coul-SR potentials. 51M inhibitor has shown higher Coul-SR potential with Arg483, and LJ-SR potential with Arg415. 1VV ligand initially shown strong H-bond and π-σ contacts with Arg415 but these interactions were not sustained for long, and resulted in poor LJ-SR or Coul-SR potential. The amount of LJ-SR and Coul-SR potentials could contribute to the estimation of interaction energy with other residues such as Ala556, Asn414, Gly530, Tyr525, Tyr334, Tyr572, Ser363, Ser508, and Ser555, which would account for stability as well as potency.

PCA is the most common method to reveal the critical motions of a protein. In all four simulations (Figure 7, Figure S6) it was observed that the protein remained relatively stable throughout the MD simulation except at the second blade and C-terminal region. The results of PCA are in line with the RMSD plots (Figure 3 and Figure 4) of the protein, where we can see a steady-state RMSD of the backbone (less than 1.25 Å) throughout the MD simulations for all simulated structures. Our PCA results are similar to Cheng et al. [39]. They have performed the MD simulations on mutated keap1 protein and found significant moments in the second propeller blade of kelch domain.

Meanwhile, calculating the FEL provides intimate details about protein folding and energy minima of the protein–ligand complex. The FEL is dependent on the simulation setup, time length, temperature, and the number of trajectories run. 5FNU, 4XMB, and 5CGJ complexes showed well-pronounced energy minima reflecting the stability of the protein–ligand complex. However, in the case of 4L7B_1VV complex, the ligand was not stable, and during simulation the ligand flipped inside the binding pocket. From 2D FEL analysis, it is clear that the ligand followed a long path to achieve its energy minima.

Then, we performed US simulations to calculate the binding free energy of ligands. The obtained binding free energies followed the potencies of the studied compounds (except 4XMB). Micromolar active inhibitors (51M and 1VV) showed the lower binding free energies and nanomolar active inhibitors (41P and L6I) displayed higher binding free energies. Obtained binding free energies for 5CGJ_51M and 4L7B_1VV complexes were lower because these inhibitors had lower interaction potential with binding site residues. 4XMB_41P complex shows a higher binding free energy (IC_50_ = 61nM) than 5FNU_L6I (IC_50_ = 15nM) because it occupies all five pockets of the binding site more compactly. In addition, structural components such as amide, phenols, sulphonyl groups resulting in a higher number of H-bonds interactions with binding site residues (Figure 6, Figure S5). This could be the probable reasons for the 4XMB structure to have a higher binding free energy than 5FNU, despite its lesser potency than 5FNU.

In this study, we employed direct Keap1-Kelch domain X-ray crystal structures to identify several crucial residues using MD simulations and other techniques. Literature review suggests that a few tens of nanoseconds of all-atom MD simulation brought valuable insights into the molecular mechanism of protein–ligand interactions [25,26,27,28]. With the ever-increasing computational facility and hardware advancement, achieving the time scales to several microseconds to milliseconds is now possible. Nevertheless, simulations of larger atomic size systems for longer periods are computationally very expensive to obtain the complete details of thier conformational characterization. Ideally, intimate details and general properties of a protein should be achievable in a computationally manageable way.

Based on our study, we can emphasize the use of MD and US simulation techniques for prioritizing the sub-pockets of keap1-Nrf2 interactions. A single change in substitution could show drastic changes in the binding site orientation of a ligand. To avoid this problem, unoccupied binding sub-pocket can be fill using substituent having higher LJ-SR and Coul-SR energies for residues surrounding targeted sub-pocket. Higher interaction energy will give stable RMSD. In addition, binding free energy from US simulations will reflect the strength of protein–ligand interactions. So an integrated approach is beneficial for the selection of bio-isosteres and ultimately obtaining more drug-like scaffolds.

Overall, a combined approach of docking, MD simulation, interaction energy calculations, FEL analysis, and binding free energy estimations is useful for design and development of potent PPI inhibitors. Using several analysis techniques and various drug design approaches (virtual screening, fragment-based, and de novo design), researchers could decrease the failure and accelerate the drug design process.

## 4. Materials and Methods

### 4.1. Protein–Ligand Complex Selection

The 5FNU, 4XMB, 5CGJ, and 4L7B crystal structures were downloaded from the RCSB protein database. The selected structures were prepared using Discovery Studio client software (BIOVIA, San Diego, CA, USA) [36]. 2D- and 3D-interactions of the crystal structures analyzed by Discovery Studio client and are shown in Figures S2 and S3.

### 4.2. Molecular Docking Study

Docking was performed by using Maestro interface in Schrödinger software (Schrödinger LLC, NY, USA). Ligands structure files were taken from the RCSB website. Protein structures were fixed by adding missing side-chain and neutralized at pH 7.4. Water was removed with less than one hydrogen bond, and only hydrogens minimized with an OPLS_2005 force field. All ligands were prepared using LigPrep tool with ligands protonated at neutral pH using Epik, and energetically minimized with an OPLS_2005 force field. Individual crystal structures were used for receptor grid generation. Grid box kept at the center of the workplace ligand. Docking was performed using the Glide standard precision (SP) method, keeping the ligand sampling as flexible. Top ten docked poses were obtained. Ligand conformation with highest binding scores were alien over original X-crystal structures and RMSD were analyzed using PyMol software (Schrödinger LLC, NY, USA) [www.pymol.org].

### 4.3. Molecular Dynamic (MD) Simulation

The four selected protein–ligand complexes were prepared using Discovery Studio with the default parameters further used for MD simulation. The first 50 ns simulation was carried out for each complex using Gromacs version 5.0.6 [37]. The simulation was further extended to 100 ns. The simulation system was set up using the CHARMM-GUI web-based graphical interface [40,41]. The CHARMM General Force Field (CGenFF) program version 1.0.0 (University of Maryland, Baltimore, MD, USA) was used for ligand preparation, with the CHARMM36m force field used for system preparation. CGenFF consider partial atomic charge for parameterization of ligand (http://docs.silcsbio.com). Charges were determined using an extended bond-charge increment scheme [42]. A water box of cubic shape and edge distance 20 Å used. Ionic NaCl solution of 0.15 M concentration was used to neutralize the solvated system (Figure 2). The number of water molecules required to explicitly hydrate the system and the number of Na^+^ and Cl^−^ ions needed to make each system electroneutral are given in Table S3. Once the system neutralized, energy minimization was performed to remove steric clashes. These energy-minimum systems were equilibrated in six steps by reducing force constant gradually and thermalized (in the NVT ensemble) at 300 K and pressurized (in the NPT ensemble) at 1 bar each for 100 ps. The well-equilibrated systems were then simulated using Gromacs software installed on supercomputing facilities provided by Korea institute of science and technology information (KISTI). The output of the simulation was first re-centered, and the trajectories analyzed by VMD software (University of Illinois at Urbana-Champaign, Urbana, IL, USA) [43]. Several analysis methods were employed for trajectory analysis, including RMSD calculation, interaction energy calculation, PCA, and free energy landscape determination. PCA analysis was carried out using the ‘gmx covar’ utility on backbone atoms. We projected the first three PCs as eigenvectors using the ‘gmx anaeig’ utility of Gromacs. This projected eigenvectors shown by the ‘Modevectors plugin’ of PyMol software (Schrödinger LLC, NY, USA). 

### 4.4. Umbrella Sampling (US) Simulation

The average structure from the last 10 ns of each simulation was selected for further US study. The initial system was prepared by using Gromacs software The protein–ligand complex was first made parallel to the *z*-axis. To pull the ligand along the *z*-axis by a 5.0 nm distance, a box was constructed with a *z*-axis length of 12 nm, as shown in Figure 9. The prepared box was solvated, neutralized, minimized and equilibrated at a temperature (NVT) and specific pressure (NPT) similar to the previous MD simulation step. The US simulation began with the center-of-mass-pulling method. The ligand was pulled from the protein pocket towards the solvent bulk over the course of 300 ps by using a 1000 kJ/(mol*nm) force. The ligand pulled at the rate of 0.01 nm per ps. During this simulation, snapshots were saved at each ps, so in total 300 configurations were generated from the pulling simulations. Several configurations were used as starting configurations for each US simulation, where each was independently simulated by performing NPT equilibration for 100 ps. Next, 1 ns MD run was performed for each individual configuration. Using the outputs from this US, the potential mean force (PMF) was calculated using the weighted histogram analysis method (WHAM), included in Gromacs as the gmx_wham utility. The generated PMF graphs show the force value in kcal/mol on the *y*-axis, which is the force needed to pull the ligand from the binding pocket, and the corresponding distance pulled during the simulation indicated on the *x*-axis. To attain equilibration, we used in total 117, 97, 103, and 84 configurations for 5FNU, 4XMB, 5CGJ, and, 4L7B, respectively. The binding free energy (ΔG) was calculated for each ligand by taking the difference between the plateau region of the PMF curve and the energy minimum of each simulation. In total, we performed 401 ns of US simulation.

## 5. Conclusions

In this study, we performed MD simulation of four X-ray structures of keap1-Nrf2 inhibitors. We identified Tyr334, Arg415, Ser508, Tyr525, Tyr572 to be crucial for hydrophobic interactions, and Ser363, Arg483, Ser508, Gly530, Ser555, and Ser602 significant for the electrostatic interactions. Obtained binding free energies followed the potencies of the studied compounds. Taken together, essential interaction networks and individual residual energetic contributions can be further exploited to design and develop novel potent Keap1-Nrf2 PPI inhibitors.

## Figures and Tables

**Table 1:** Four crystal structures selected for molecular dynamic (MD) and umbrella sampling (US) study.

Protein Structure	Ligand Name	Resolution	Activity (IC_50_) FP Assay	MD Simulation (ns)	
				First half	Second half
4L7B	1VV	2.41Å	2.3 µM	50	100
5CGJ	51M	3.36Å	0.14 µM	50	100
4XMB	41P	2.43Å	61 nM	50	100
5FNU	L6I	1.78Å	15 nM	50	100

**Table 2:** Residue interaction energy analyses of four complexes for 50 ns MD simulation.

Residue	5FNU (L6I) (IC_50_ = 15 nM)		4XMB (41P) (IC_50_ = 61 nM)		5CGJ (51M) (IC_50_ = 0.14 µM)		4L7B (1VV) (IC_50_ = 0.75 µM)	
	LJ-SR kJ/mol	Coul –SR kJ/mol	LJ-SR kJ/mol	Coul-SR kJ/mol	LJ-SR kJ/mol	Coul-SR kJ/mol	LJ-SR kJ/mol	Coul-SR kJ/mol
**Tyr 334**	−22.08	−5.96	−15.32	−4.14	−21.41	−3.35	−17.44	−18.71
**Ser 363**	−7.42	−10.45	−7.73	−22.68	−5.54	−6.43	−2.09	0.41
**Gly 364**	−6.53	−3.44	−9.31	−1.29	−6.64	−0.07	−1.39	0.49
**Arg 380**	−2.01	1.01	−6.81	3.63	−1.30	0.55	−5.10	−2.19
**ASN 382**	−0.75	0.067	−0.03	−0.02	−1.82	0.36	−3.25	−0.92
**Asn 414**	−1.73	0.32	−4.15	−22.40	−1.70	0.11	−1.07	−4.11
**Arg 415**	−25.36	4.019	−24.87	−43.66	−29.077	9.19	−7.44	−10.12
**Ile 461**	−5.343	−0.55	−7.22	−2.52	−6.113	−0.005	−8.80	−0.07
**Gly 462**	−5.74	1.01	−6.79	−1.32	−6.46	−0.04	−0.84	0.37
**Phe 477**	−8.20	−2.55	−4.47	0.67	−8.63	−4.16	−0.03	0.03
**Arg 483**	9.59	−173.22	−5.48	5.75	−1.85	−39.09	−0.39	0.182
**Ser 508**	−3.72	−59.40	−7.66	−18.12	−10.13	−11.05	−0.54	0.037
**Gly 509**	−6.27	1.20	−7.95	0.25	−8.24	0.75	−0.98	0.311
**Tyr 525**	−34.91	−9.86	−19.39	−4.24	−8.88	−0.47	−1.69	−0.28
**Gly 530**	−3.72	−29.59	−6.67	−2.14	−0.52	0.44	−0.57	−0.188
**Ser 555**	−6.00	−13.48	−9.13	−2.91	−5.11	0.70	−1.53	−0.34
**Ala 556**	−11.81	1.34	−15.60	−4.18	−14.91	−1.13	−3.28	−0.01
**Tyr 572**	−14.14	−4.34	−16.75	−1.79	−10.79	−0.60	−13.48	−2.27
**Phe 577**	−3.03	−1.63	−8.85	−1.12	−7.69	0.78	−8.41	−1.38
**Ser 602**	−4.40	−29.12	−10.02	−17.11	−9.37	−21.23	−3.26	−3.46
**GLY 603**	−8.22	−0.26	−9.38	−0.64	−9.59	0.17	−1.84	0.39
**Total**	−171.79	−334.88	−203.58	−139.98	−175.77	−74.57	−83.42	−41.82
**Total LJ_SR and Coul-SR**	−506.67		−343.56		−250.34		−125.24	

**Table 3:** Binding free energy obtained using umbrella sampling simulation.

Protein Structure	Ligand Name	Resolution	Year	Activity (IC_50_)	Binding Free Energy (kcal/mol)
4L7B	1VV	2.41 Å	2013	0.75µM	−4.35
5CGJ	51M	3.36 Å	2015	0.14µM	−4.55
4XMB	41P	2.43 Å	2015	61nM	−13.48
5FNU	L6I	1.78 Å	2016	15nM	−9.80

## Supplementary Materials

The following are available online at https://www.mdpi.com/1420-3049/24/22/4085/s1. See supplementary Word file.

## Abbreviations

MD	Molecular dynamic
US	Umbrella sampling
PPI	Protein–Protein interactions
RMSD	Root mean square deviation
PMF	Potential of mean force
Coul-SR	Coulombic potential short-range
LJ-SR	Lennard–Jones potential short-range
PCA	Principal component analysis
H-bond	Hydrogen bond
ns	Nanosecond
µs	Microsecond
nm	Nanometer
ps	Picosecond
nM	Nanomolar
µM	Micromolar
NVT	Constant number of atoms, volume, and temperature
NPT	Constant number of atoms, pressure, and temperature
