# Supplementary material for: Investigation of Molecular Details of Keap1-Nrf2 Inhibitors Using Molecular Dynamics and Umbrella Sampling Techniques
Source: Molecules. 2019 Nov 12;24(22):4085. doi: 10.3390/molecules24224085 (PMC6891428; doi:10.3390/molecules24224085)
Supplement: Supplementary file 1 [file molecules-24-04085-s001.pdf]

Supplementary Material

Investigation of Molecular Details of Keap1-Nrf2 Inhibitors using Molecular Dynamics and Umbrella Sampling Techniques

Ashwini Machhindra Londhe [1,2], Changdev Gorakshnath Gadhe [1], Sang Min Lim [1,2] and Ae Nim Pae [1,2,*]

[1] Convergence Research Center for Diagnosis, Treatment, and Care system of Dementia, Korea Institute of Science and Technology, Seoul, Korea; londheash@gmail.com/615502@kist.re.kr (A.M.L); gadhe.changdev@gmail.com (C.G.G.); smlim28@kist.re.kr (S.M.L)

[2] Division of Bio-Medical Science & Technology, KIST School, Korea University of Science and Technology, Seoul, Korea

* Correspondence: anpae@kist.re.kr; Tel.: +82-010-5013-5074

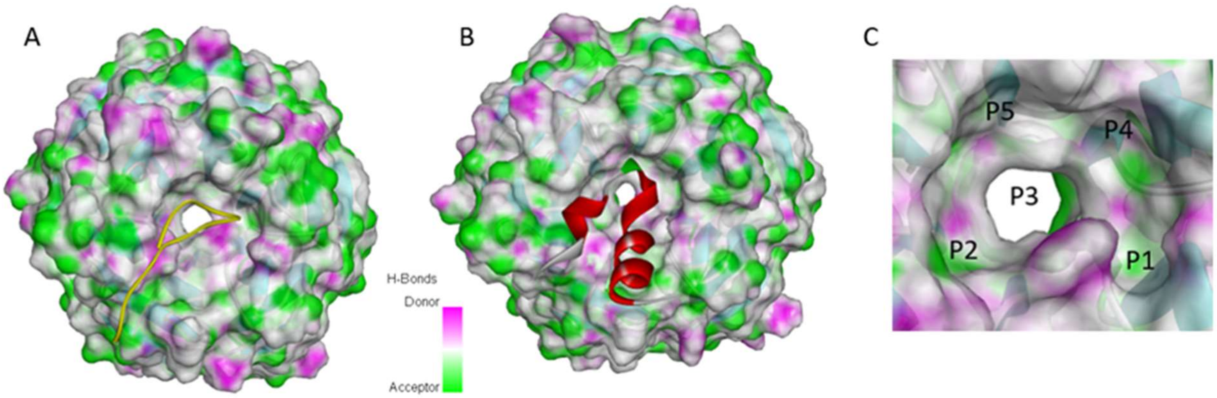

Figure S1. Keap1-kelch domain and Nrf peptide, protein-protein interactions (PPI). DxETGE motif (PDB ID: 2FLU) and DFGex motif (3WN7) of NRF2 peptide binds to Kelch domain of Keap1 protein shown in (A) and (B) respectively. (C) Most of the Keap1-Nrf2 PPI inhibitors which binds at kelch domain occupies completely or partially the five subpockets (P1-P5) of binding site.

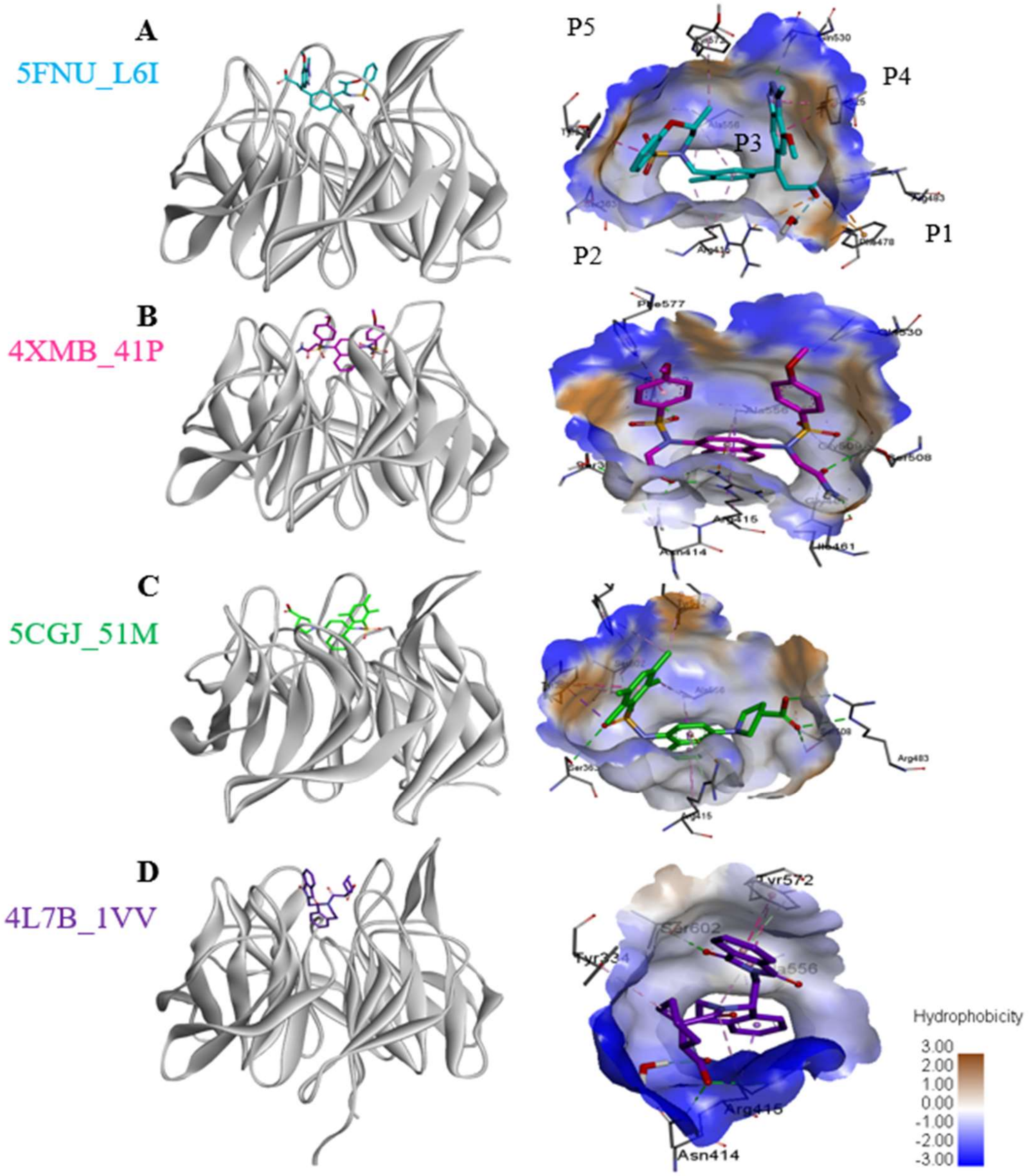

Figure S2. 5FNU, 4XMB, 5CGJ, and 4L7B crystal structures selected for the umbrella sampling study. Ligand-protein complex and 3D interaction inside binding pocket is (hydrophobic surface) shown. Co-crystal ligands L6I, 41P, 51M, 1VV shown in cyan, magenta, green and purple color respectively. Interactions such as H-bonds, electrostatic, π-π stacking, π-σ, π –sulfur, and π-alkyl/alkyl shown in green, orange, dark pink, purple, yellow and light pink color respectively. Water molecule within binding pockets shown in stick format.

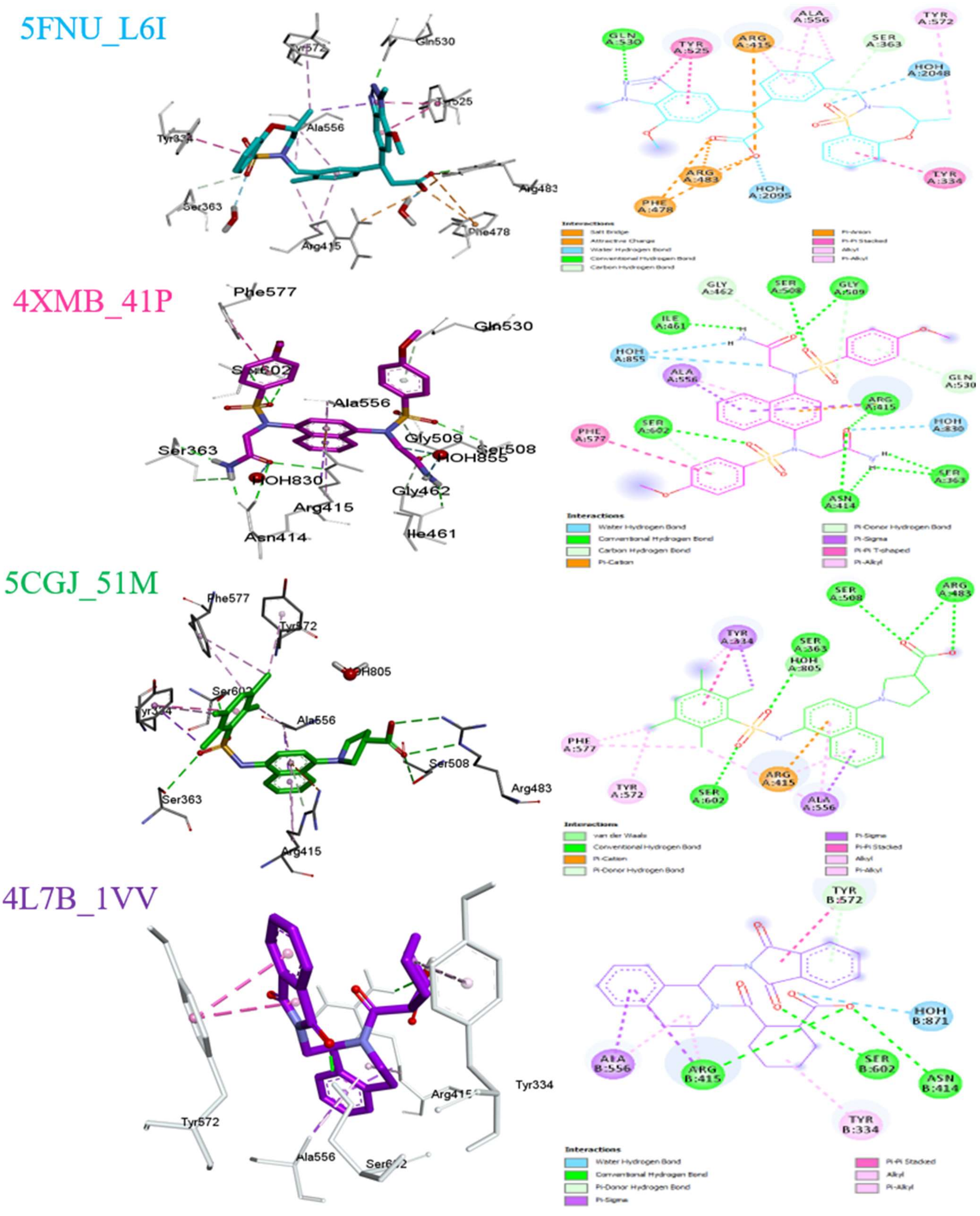

Figure S3. 2D and 3D interaction diagram of selected four crystal structures.

1 Interaction before MD simulation

Kelch domain binding site is consist of five sub pockets P1-P5, shown in **Figure S1C**.

5FNU

The L6I ligand with 15 nM activity held inside pocket by several interactions such as π-stacking contacts of a phenyl-benzo-oxathiazepin ring in P5 pocket **Figure S2-S3**. Phenyl ring has π-π stacking interactions with Tyr334, sulphonyl oxygen has week carbon-H-bond with Ser363, and methyl substitution has π –alkyl contact with Tyr572. Central phenyl group with methyl substitution has hold inside P3 pocket by Ala556 and Arg415 mediated hydrophobic interactions. 7-methoxy-1-methyl-1H benzo[d][1,2,3] triazole-5-yl group fixed inside P4 pocket by forming strong π-π stacking bond with Tyr525 and Gly530 mediated H-bond with Triazole nitrogen. The P1 pocket acid group has electrostatic and H-bond interactions with Arg415, Arg483 and phe478. Inside P1 pocket one water molecule is trapped showing conventional H-bond/water H-bond with an acid group of ligand.

4XMB

In case of 4XMB crystallized ligand 41P, amide groups occupied the P1 and P2 pocket by a secure network of the H-bond with Ser363, Asn414, Arg415, Ile461, and Gly509 **Figure S2-S3**. P3 pocket naphthalene ring is sandwich between Ala556, and Arg415 by π-σ, π –alkyl, π-cation (electrostatic) interactions. Among two hydroxyl phenyl group, one group fixed inside P5 pocket showing π-stacking contact with phe577. Second hydroxyl phenyl group occupied the P4 pocket, but its interactions with surrounding residues were not visible by discovery studio visualizer. However, this missing interactions then visualized during MD simulation trajectories (**Figure S7)**. Sulphonyl oxygen involved in H-bond interactions with Ser508, Ser602 along with hydrophobic interactions of Gly509.

5CGJ

The 51M ligand in the 5CGJ crystal structure is small ligand having an acid group that makes a strong H-bond with Ser508 and Arg483 at P1 pocket **Figure S2-S3**. P3 pocket naphthalene ring bounded by π-σ, π –alkyl, and π-cation contacts with Ala556 and Arg415. Sulphonyl oxygen involved in an H-bond with Ser363 and Ser602. P5 pocket tetra-methyl phenyl group complex established a network of π-σ, π-π stacking, and π –alkyl contacts with Tyr334, Phe577, and Tyr572. This molecule has not occupied the P4 pocket.

4L7B

Second micromole active molecule 1VV has occupied only P2, P3, and P5 pocket **Figure S2-S3**. Acid group inserted inside P2 pocket make H-bond with Asn414, Arg415, and one H-bond with a water molecule. Further, cyclohexane ring shown π–alkyl contact with Tyr334. P3 pocket dihydro-isoquinoline has π-σ and π –alkyl contact with Arg415, Ala556. Tyr572 from P5 pocket holds the isoindole ring by strong π-π stacking interactions. Oxygen group of this ring form H-bond with Ser602.

2 Molecular Docking simulation

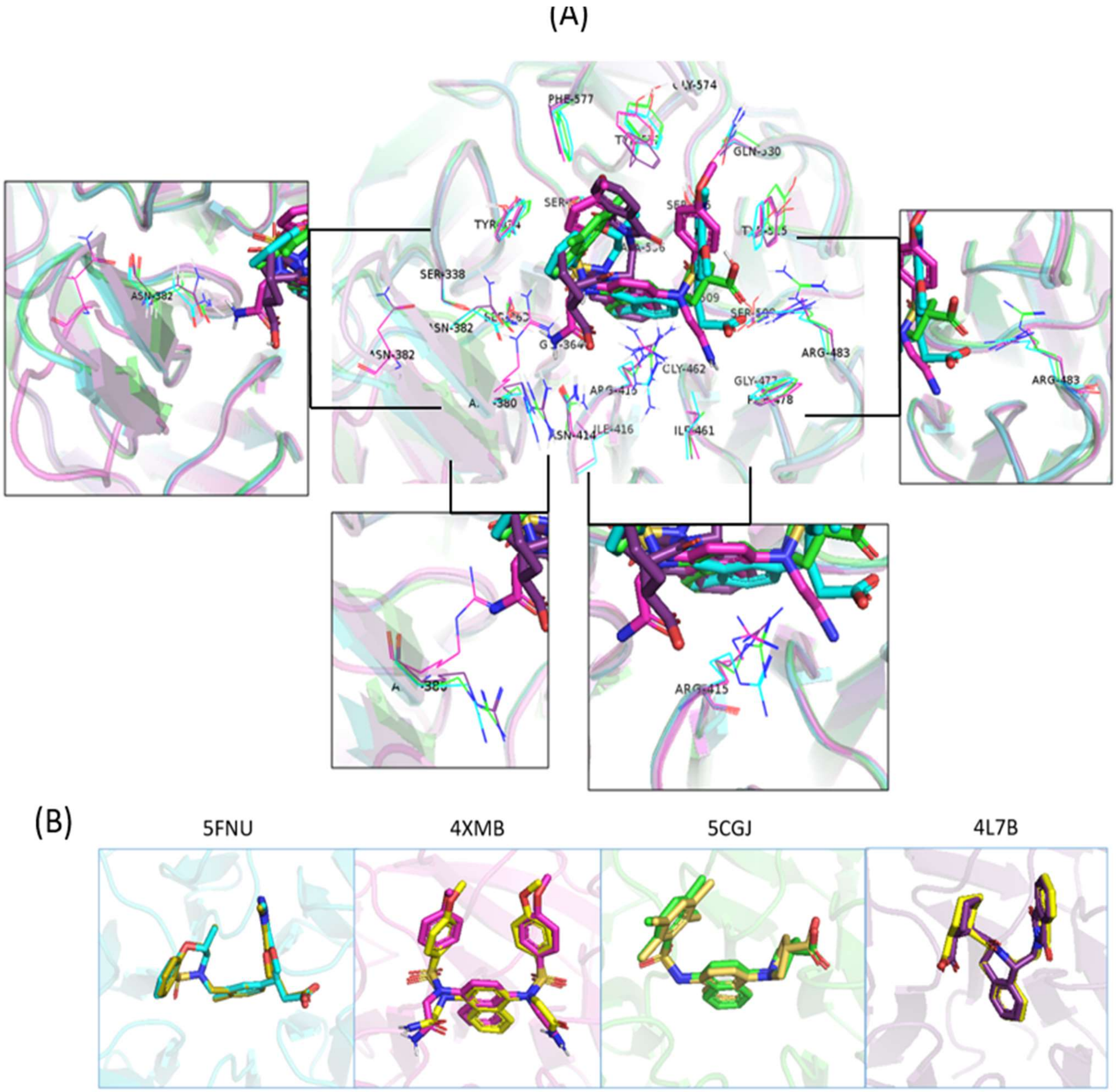

Figure S4. Docking study results. (A) 5FNU (cyan), 4XMB (magenta), 5CGJ (green) and 4L7b (purple) crystal structures aligned over each other. Ligand binding site residue (4Å) shown in line format. The significant difference observed in the orientation of Asn382, Arg380, Arg415, and Arg483. (B) The topmost binding pose of each docked ligands (yellow) shown over the respective cocrystal structure.

Table S1. Molecular docking study results

Crystal structure	RMSD (Å)	Docking score
4L7B	0.25	-7.88
5CGJ	0.28	-7.38
4XMB	1.19	-11.48
5FNU	0.24	-10.48

3 Molecular dynamic Simulation

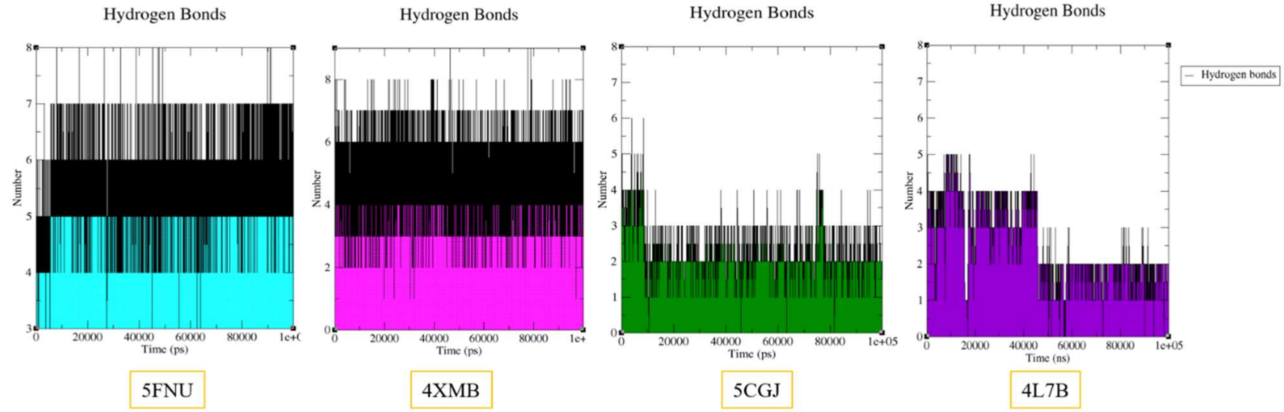

Figure S5 Assessment of number of H bonds during 100 ns MD simulations.

Table S2. Residue interaction energy analyses of four complexes for 100 ns MD simulation.

Residue	5FNU (L6I) (IC_{50} = 15nM)		4XMB (41P) (IC_{50} = 61nM)		5CGJ (51M) (IC_{50} = 0.14μM)		4L7B (1VV) (IC_{50} = 0.75μM)	
	LJ-SR kJ/mol	Coul -SR kJ/mol	LJ-SR kJ/mol	Coul-SR kJ/mol	LJ-SR kJ/mol	Coul-SR kJ/mol	LJ-SR kJ/mol	Coul-SR kJ/mol
Tyr 334	-21.75	-5.65	-15.89	-4.16	-21.23	3.4	-11.07	0.26
Ser 363	-6.89	-9.75	-8.43	-19.04	-5.59	-5.88	-5.12	-1.51
Gly 364	-6.26	-3.18	-9.43	-1.21	-6.83	-0.39	-4.02	0.35
Arg 380	-1.62	0.93	-7.44	3.43	-1.32	0.51	-4.19	-2.24
Asn 382	-0.94	0.06	-0.02	-0.01	-1.16	0.08	-0.1	0.01
Asn 414	-1.5	0.3	-3.61	-23.36	-1.79	0.06	0.63	-20.48
Arg 415	-18.49	0.41	-24.63	-40.83	-28.05	8.53	-22.24	-81.39
Ile 461	-5.4	-0.6	-6.68	-0.81	-6.19	-0.04	-5.53	-0.98
Gly 462	-5.68	0.93	-6.21	-1.97	-6.67	-0.24	-3.8	-3.82
Phe 477	-0.83	-0.67	-0.71	-0.55	-0.8	0.37	-0.57	0.65
Arg 483	12.16	-178.96	-5.58	6.08	-1.83	-38.54	-1.85	-0.59
Ser 508	-1.94	-62.54	-8.03	-19.81	-10.22	-10.21	-4.55	-0.47
Gly 509	-6.38	-1.39	-8.69	0.14	-8.18	0.81	-4.54	0.94
Tyr 525	-34.34	-9.46	-19.04	-4.1	-8.81	-0.54	-9.53	-0.39
Gly 530	-3.87	-28.24	-5.74	-3.09	-0.56	-0.51	-0.86	0.65
Ser 555	-5.91	-13.6	-10.25	-8.14	-5.17	0.83	-4.34	-1.65
Ala 556	-12.35	1.41	-16.15	-4.51	-14.56	-1.18	-7.75	0.98
Tyr 572	-14.47	-4.02	-17.11	-2.16	-10.6	-0.55	-10.21	-0.83
Phe 577	-3.41	-1.83	-8.75	-1.18	-7.58	0.78	-3.55	0.45
Ser 602	-5.03	-29.31	-9.86	-17.4	-9.48	-21.14	-6.04	-4
Gly 603	-7.99	-0.29	-9.26	-0.63	-9.42	-0.17	-4.49	0.58
Total	-152.89	-345.45	-201.51	-143.31	-166.04	-64.02	-113.72	-113.48
Total LJ_SR and Coul-SR	-498.34		-344.82		-230.06		-227.2	

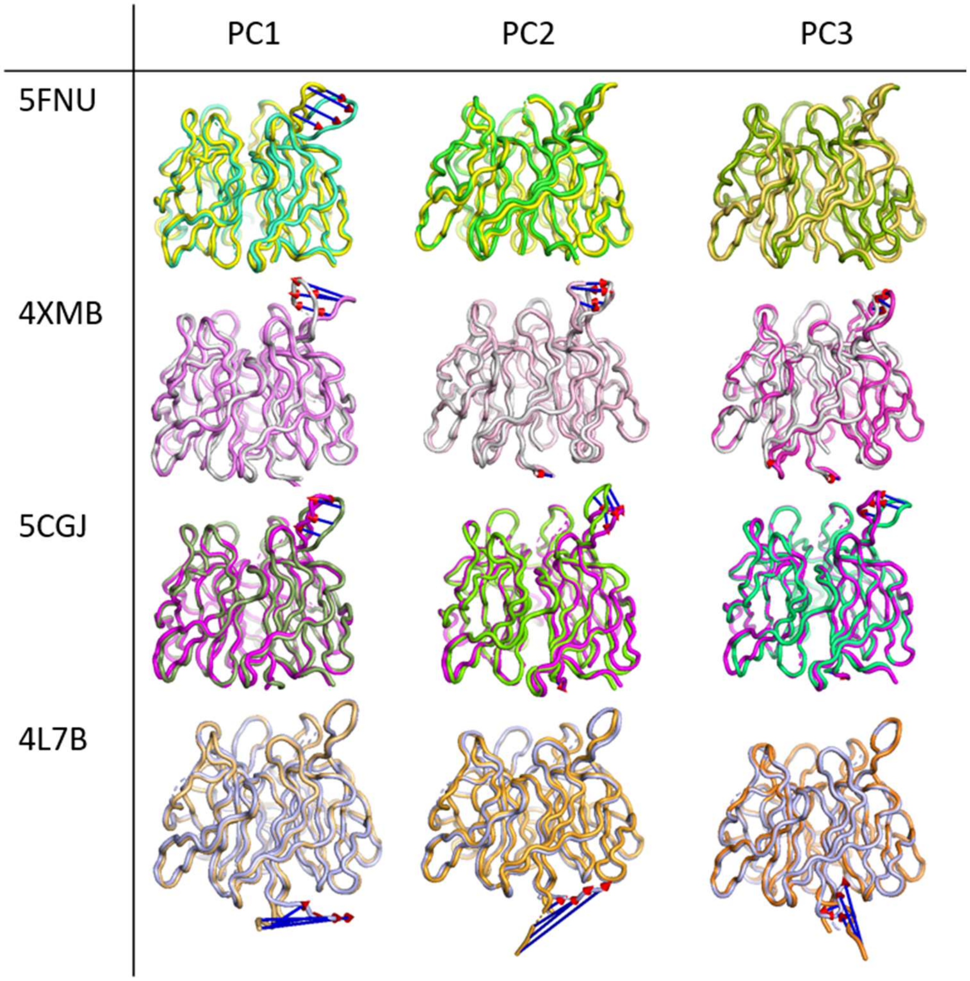

Figure S6 Principal component analysis. Eigenvectors from 100 ns MD trajectories depicting the movement of Cα atoms in four crystal structures. The initial position of the protein backbone shown in wheat, gray-white, pink, and green for 5FNU, 4XMB, 5CGJ, and 4L7B, respectively. The movements of flexible parts of the protein shown with a red-head and blue-tail containing arrow.

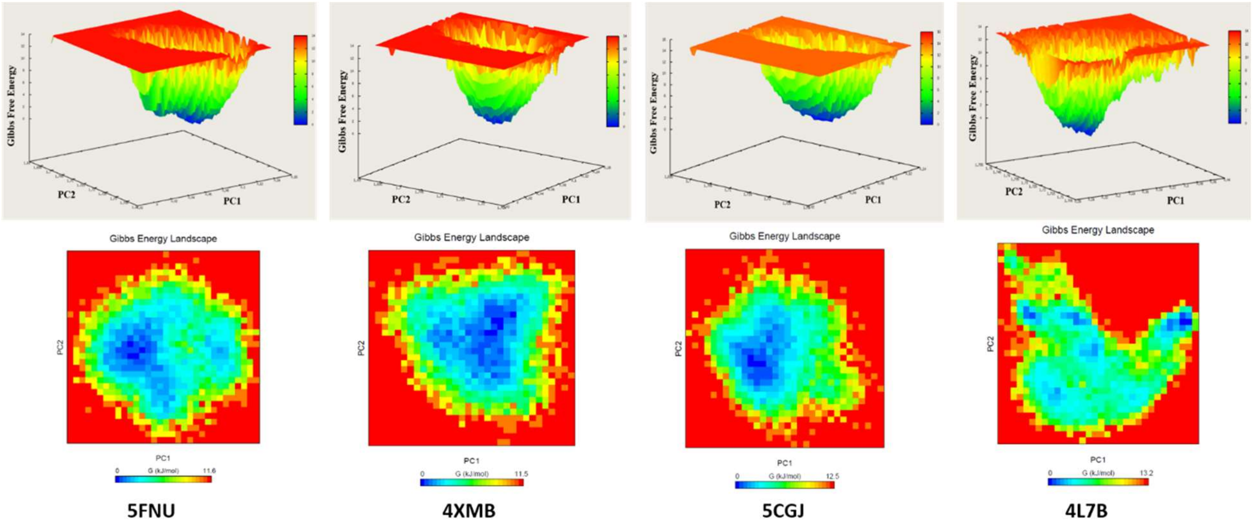

Figure S7 Free energy landscapes for four crystal structures from 100 ns MD simulation. 2D and 3D graphs projected on the first two principal components (PC1+PC2). Blue spots indicate the energy minima.

Table S3. Molecular dynamic simulation system prepared for four protein-ligand complexes.

	**5FNU**	**4XMB**	**5CGJ**	**4L7B**
Ligand Name	**L6I**	**41P**	**51M**	**1VV**
Protein atom	2862	2845	2875	2896
Ligand atom	48	60	40	41
Water molecule	22038	21222	21242	21528
Na ion	68	64	60	62
Cl ion	60	58	53	54
NPT	1 bar	1 bar	1 bar	1 bar
NVT	300K	300K	300K	300K
